# Supplementary material for: A fault-intrusion-tolerant system and deadline-aware algorithm for scheduling scientific workflow in the cloud
Source: PeerJ Comput Sci. 2021 Nov 2;7:e747. doi: 10.7717/peerj-cs.747 (PMC8576554; doi:10.7717/peerj-cs.747)
Supplement: Supplemental Information 6 [file peerj-cs-07-747-s006.zip › Myproject1.1/docs/org/cloudbus/cloudsim/ParameterException.html]

ParameterException


JavaScript is disabled on your browser.


- Overview
- Package
- Class
- Use
- Tree
- Deprecated
- Index
- Help

- Prev Class
- Next Class

- Frames
- No Frames

- All Classes

- Summary:
- Nested |
- Field |
- Constr |
- Method

- Detail:
- Field |
- Constr |
- Method


org.cloudbus.cloudsim

## Class ParameterException

- java.lang.Object
- - java.lang.Throwable
  - - java.lang.Exception
    - - org.cloudbus.cloudsim.ParameterException

- All Implemented Interfaces:
  :   java.io.Serializable

  ---

    

  ```
  public class ParameterException
  extends java.lang.Exception
  ```

  This exception is to report bad or invalid parameters given during constructor.

  Since:
  :   CloudSim Toolkit 1.0

  See Also:
  :   Serialized Form

- - ### Constructor Summary

    Constructors

    Constructor and Description

    `ParameterException()`

    Constructs a new exception with null as its detail message.

    `ParameterException(java.lang.String message)`

    Creates a new ParameterException object.
  - ### Method Summary

    Methods

    | Modifier and Type | Method and Description |
    | --- | --- |
    | `java.lang.String` | `toString()` Returns an error message of this object. |

    - ### Methods inherited from class java.lang.Throwable

      `addSuppressed, fillInStackTrace, getCause, getLocalizedMessage, getMessage, getStackTrace, getSuppressed, initCause, printStackTrace, printStackTrace, printStackTrace, setStackTrace`
    - ### Methods inherited from class java.lang.Object

      `clone, equals, finalize, getClass, hashCode, notify, notifyAll, wait, wait, wait`

- - ### Constructor Detail


    - #### ParameterException

      ```
      public ParameterException()
      ```

      Constructs a new exception with null as its detail message.


    - #### ParameterException

      ```
      public ParameterException(java.lang.String message)
      ```

      Creates a new ParameterException object.

      Parameters:
      :   `message` - an error message
  - ### Method Detail


    - #### toString

      ```
      public java.lang.String toString()
      ```

      Returns an error message of this object.

      **Overrides:**
      :   `toString` in class `java.lang.Throwable`

      Returns:
      :   an error message


- Overview
- Package
- Class
- Use
- Tree
- Deprecated
- Index
- Help

- Prev Class
- Next Class

- Frames
- No Frames

- All Classes

- Summary:
- Nested |
- Field |
- Constr |
- Method

- Detail:
- Field |
- Constr |
- Method
